# Application of Pre-Column Labeling Liquid Chromatography for Canine Plasma-Free Amino Acid Analysis

**DOI:** 10.3390/metabo6010003

**Published:** 2016-01-12

**Authors:** Kazuo Azuma, Yoshiko Hirao, Yoshihiro Hayakawa, Yusuke Murahata, Tomohiro Osaki, Takeshi Tsuka, Tomohiro Imagawa, Yoshiharu Okamoto, Norihiko Ito

**Affiliations:** 1 Department of Veterinary Clinical Medicine, Tottori University, 4-101 Koyama-minami, Tottori 680-8533, Japan; ymurahata@muses.tottori-u.ac.jp (Y.M.); tosaki@muses.tottori-u.ac.jp (T.O.); tsuka@muses.tottori-u.ac.jp (T.T.); imagawat@muses.tottori-u.ac.jp (T.I.); yokamoto@muses.tottori-u.ac.jp (Y.O.); 2 Analytical and Measuring Instruments Division, Shimadzu Corporation, 1, Nishinokyo Kuwabaracho, Nakagyo-ku, Kyoto 604-8511, Japan; yoshikon@shimadzu.co.jp (Y.Hi.); yo-haya@shimadzu.co.jp (Y.Ha.)

**Keywords:** Plasma-free amino acid, pre-column labeling, liquid chromatography measurements, canine plasma amino acid levels, pre- and post-food intake

## Abstract

Plasma-free amino acid (PFAA) levels are a useful metric for diagnosing cancer and providing a prognosis. However, the use of analysis of PFAA levels has been limited in the veterinary medicine field. We addressed the application of liquid chromatography (LC) using a pre-column labeling technique for analysis of canine PFAA levels. This method significantly shortened the analysis time relative to conventional methods. No diurnal fluctuations were detected at 9:00 AM in most PFAA levels, and food intake increased the levels of some PFAAs, including valine, leucine, tyrosine, phenylalanine, and proline. These results indicate that LC with pre-column labeling is useful for measuring canine PFAA levels, for which time of day and interval after food intake must be taken into consideration.

## 1. Introduction

Amino acids (AAs) are important substrates for and regulators of metabolic pathways [1]. Indeed, the balance of plasma-free (PF) AAs is disrupted in various diseases including cancer [2,3,4,5]. Moreover, a link has been reported between canine cancer and PFAA levels; plasma levels of glutamine (Gln), serine (Ser), asparagine (Asn), and alanine (Ala) were lower in dogs with malignant mammary gland tumors than in healthy animals [6], while plasma levels of threonine (Thr), proline (Pro), and Ser were reduced in canine oral malignant melanoma relative to controls [7]. These results indicate that PFAA levels are a useful metric for diagnosing canine cancer and predicting prognosis.

Several methods are available for measuring AA levels, including liquid chromatography (LC), gas chromatography, and capillary electrophoresis, among others [8]. Although LC methods coupled with optical detection are well established and highly reliable, post-column LC procedures are time-consuming [8]. In contrast, levels of free major AAs in plasma can be determined within 17 min by pre-column derivatization followed by reversed-phase high-performance (HP) LC [9]. The shortened analysis time makes this method suitable for routine investigation of large sample sets [9]. It is posited that one reason why analysis of AA doses has not expanded in veterinary clinical medicine is their complication and the long time they require.

PFAA levels in humans are influenced by many factors, including diurnal and hormonal fluctuations and the food ingested [10,11,12,13]. To our knowledge, however, there are no reports describing the effects of diurnal fluctuations and food intake on canine PFAA levels via a pre-column LC method. The aim of this study is to evaluate the efficiency of the pre-column LC method for canine PFAA analysis and investigate the effects of diurnal fluctuations and food intake which affect PFAA levels.

## 2. Results and Discussion

### 2.1. Diurnal Fluctuations in Canine PFAA Levels Measured with Pre-Column Labeling LC

**Figure 1:**
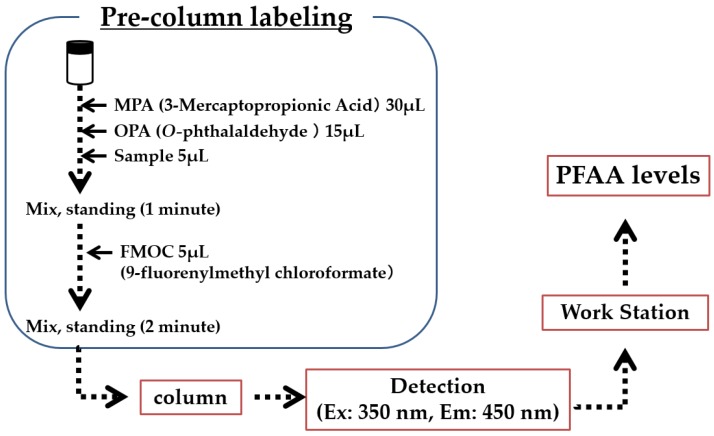
The schema of pre-column labeling method.

Table 1 indicates characteristics of dogs included in this study. All 20 AAs in canine plasma were separated within 14 min with a cycle time of 27 min, as seen in the chromatographs (Figure 1). Figure 1 shows chromatograms of (a) an AA standard and (b) a canine plasma sample. PFAA levels after deproteinization were under 250 μmol/L. There were no differences in PFAA levels across experimental days.

**Table 1:** Characteristics of dogs included in this study.

No.	Sex	Age (Years)	Body Weight (kg)
1	Male	4	12
2	Male	11	11
3	Male	9	13
4	Female	9	11
5	Female	5	10

Post-column labeling techniques are sufficiently accurate for measuring canine PFAAs [14]. However, a disadvantage of this method is that it is time-consuming. For example, in our previous report, separation required 110 min, with a cycle time of 135 min. The pre-column method used here shortened the analysis time by a considerable margin. PFAA levels are generally constant within a certain range [10,11,12].

**Figure 2:**
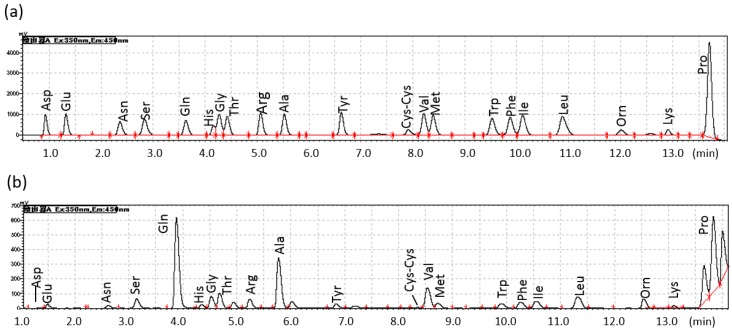
Representative chromatograms of (**a**) an AA standard and (**b**) a canine plasma sample. The concentration of AAs in the standard solution is 250 μmol/L.

**Figure 3:**
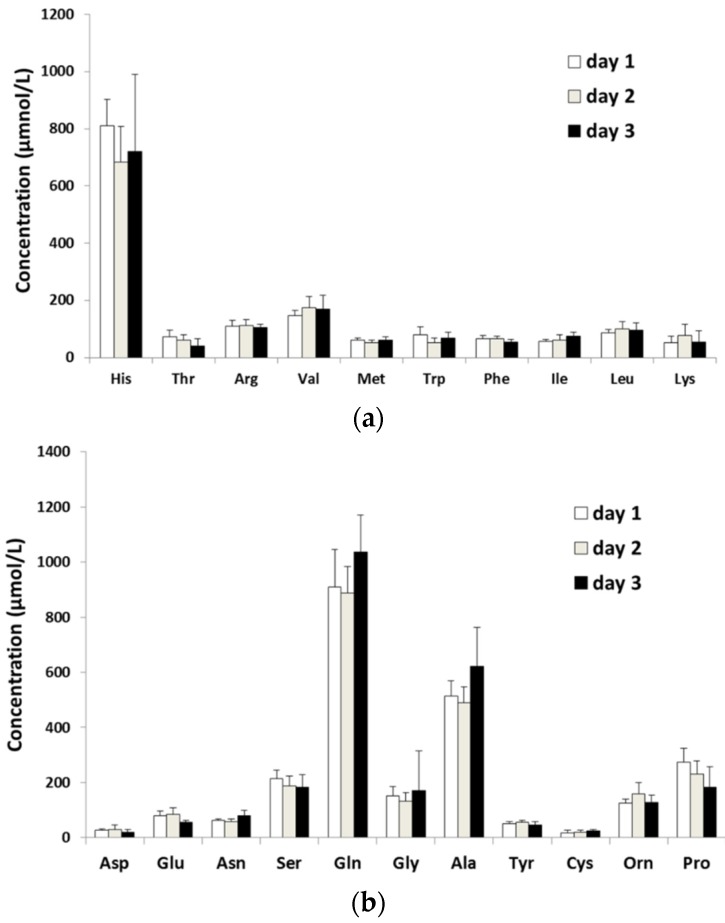
Diurnal fluctuations in AA concentration. Data are shown as mean ± SD. Results for (**a**) essential and (**b**) non-essential AAs are shown.

In the present study, there was no significant change in PFAA levels measured each day at 9:00 AM after a 14-h fast (Figure 2 and Figure 3). However, standard deviations of some PFAA levels including His, Thr, Gly, Ala, and Pro were large. Our results indicate that it is true the most canine PFAA levels exhibit diurnal fluctuations. They also indicate that some hormones affects PFAA levels [15]. In facts, sex is a factor which affects PFAA levels [15]. To understand the diurnal fluctuations of plasma His, Thr, Gly, Ala, and Pro levels, a study separated into male or female is needed. It is also reported that PFAA levels exhibit circadian rhythm in humans [16,17]. On the other hand, it is reported that serum hydroxyproline level does not affect circadian rhythm in dogs [18]. A study must be conducted focusing on circadian rhythmicity of canine PFAA levels in the future.

### 2.2. Changes in PFAA Levels after Food Intake

**Figure 4:**
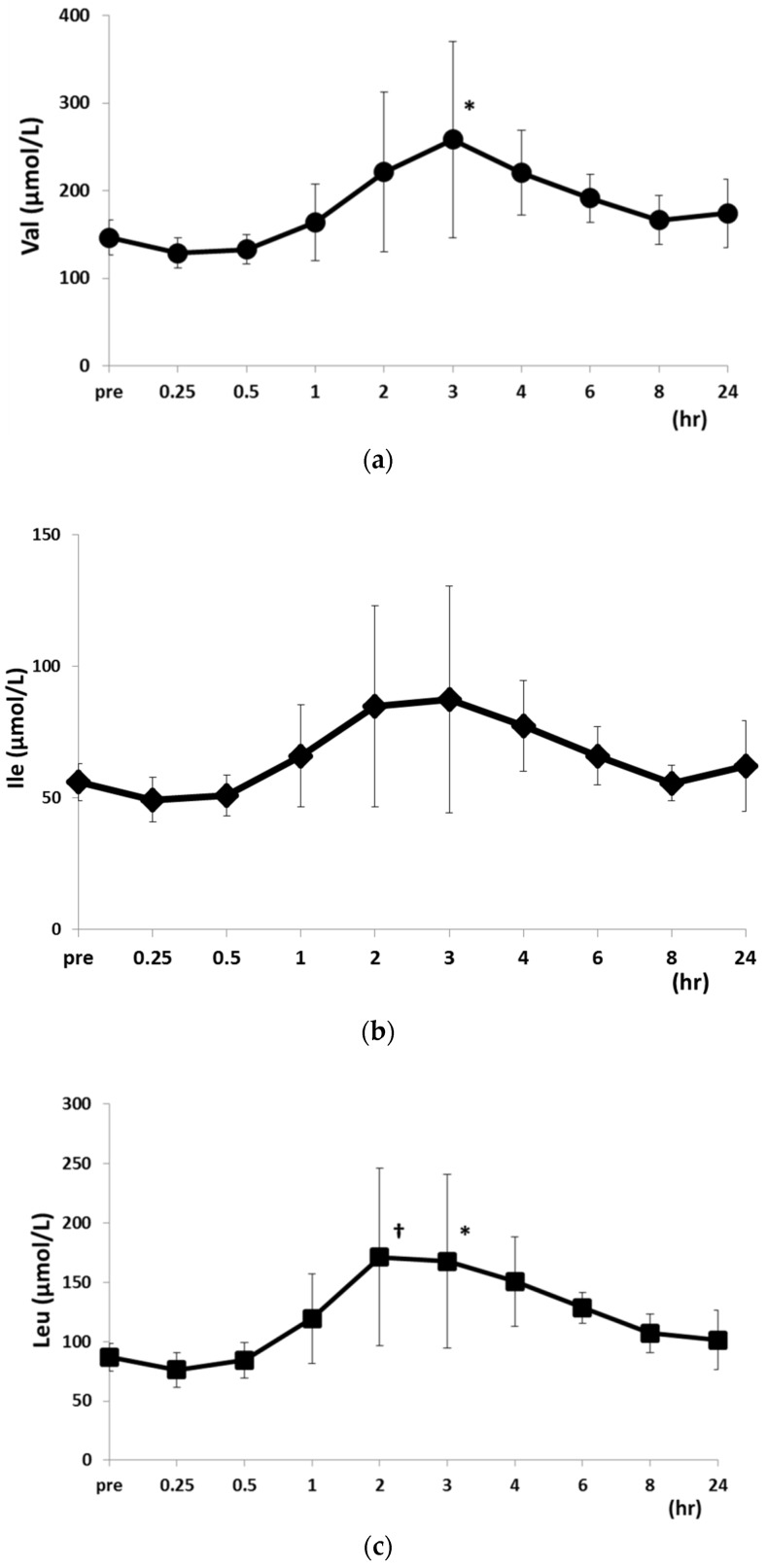
Changes in Val (**a**); Ile (**b**); and Leu (**c**) concentrations after food intake. Data are shown as mean ± SD. * *p* < 0.05 *vs.* 15 and 30 min post-food intake. ^†^ *p* < 0.05 *vs.* pre-food intake.

**Figure 5:**
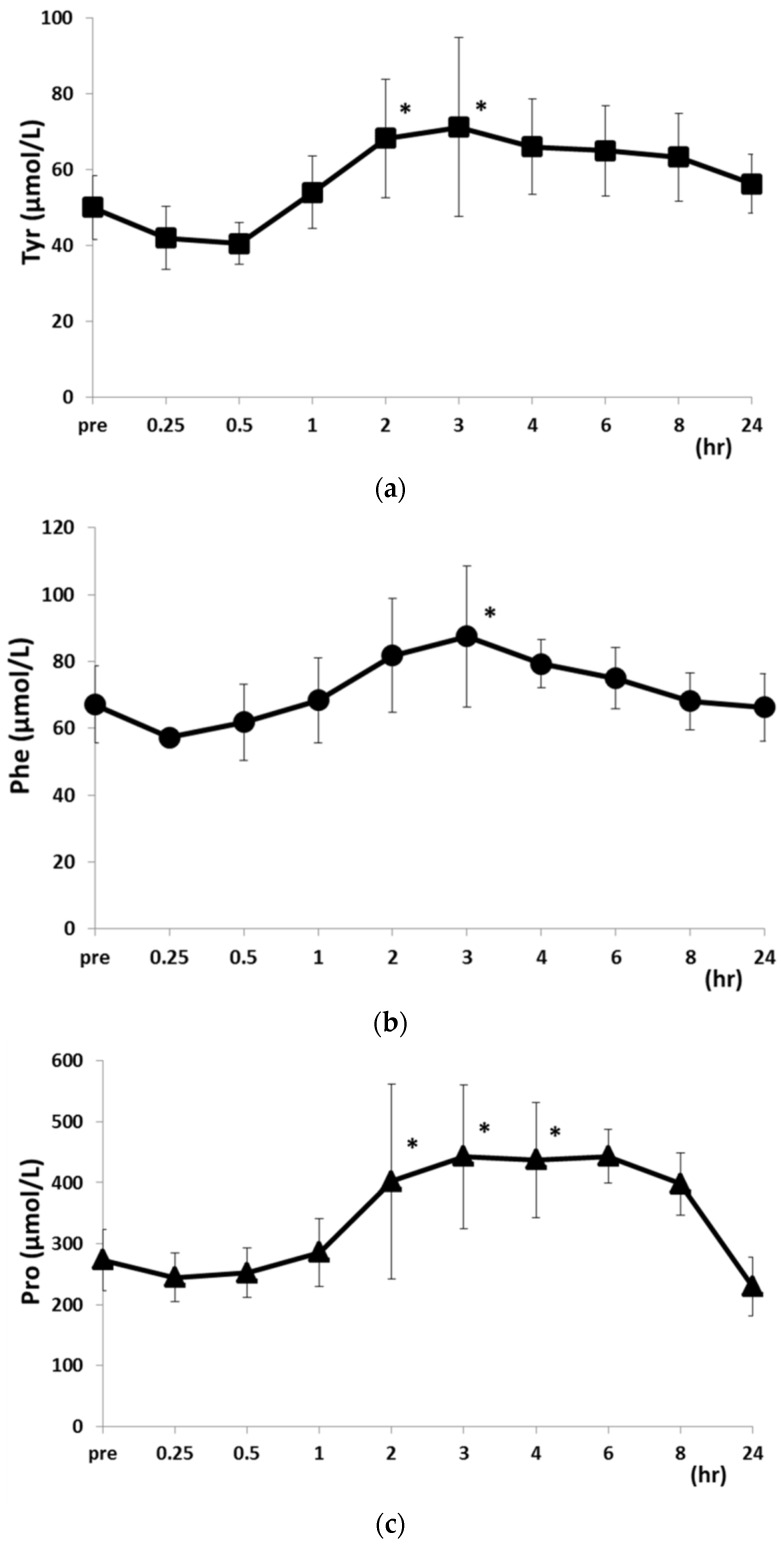
Changes in Tyr (**a**); Phe (**b**) and Pro (**c**) concentrations after food intake. Data are shown as mean ± SD. * *p* < 0.05 *vs.* 15 and 30 min post-food intake.

At 15 and 30 min after feeding, the levels of all PFAAs were equal to or slightly lower than pre-feeding levels (Table 2). At 2, 3, 4, and 6 h after feeding, plasma PFAA levels were increased relative to pre-feeding levels and those measured at the 15 and 30 min time points. At 2 h post-feeding, Leu levels were higher than before and 15 and 30 min after feeding, whereas Tyr levels were higher than at 15 and 30 min post-feeding (Figure 4). At 3 h after feeding, Val, Leu, Tyr, and Phe levels were increased as compared to those measured 15 and 30 min after feeding, and at 2, 3, and 4 h post-feeding, Pro levels were higher than 15 and 30 min post-feeding levels (Figure 5).

**Table 2:** PFAA concentrations before (Pre) and at indicated times after food intake.

	Pre	15 min	30 min		1 h	2 h	
His	803.4 ± 88.4	713.6 ± 96.9	725.2 ± 85.3		747.4 ± 99.7	844.1 ± 184.6	
Thr	66.1 ± 27.0	62.1 ± 25.4	63.9 ± 19.7		71.6 ± 10.2	96.6 ± 16.5	
Arg	110.3 ± 21.0	95.7 ± 25.7	98.2 ± 21.1		106.5 ± 15.3	123.7 ± 34.8	
Val	146.4 ± 19.8	128.8 ± 17.5	133.0 ± 16.6		164.1 ± 43.9	221.1 ± 91.2	
Met	61.4 ± 7.4	50.6 ± 8.9	55.7 ± 7.1		58.4 ± 7.7	68.3 ± 15.3	
Trp	79.7 ± 26.9	79.6 ± 28.7	88.1 ± 22.2		97.9 ± 17.1	111.6 ± 27.6	
Phe	67.1 ± 11.5	57.3 ± 1.8	61.8 ± 11.4		68.3 ± 12.7	81.8 ± 16.9	
Ile	56.1 ± 7.1	49.4 ± 8.3	51.0 ± 7.8		65.9 ± 19.4	84.8 ± 38.2	
Leu	86.8 ± 11.4	76.1 ± 14.7	84.4 ± 14.9		119.5 ± 37.7	171.3 ± 74.6	
Lys	51.3 ± 23.2	36.1 ± 7.3	35.8 ± 5.2		45.2 ± 18.4	52.6 ± 42.2	
Asp	26.8 ± 4.9	22.9 ± 8.8	23.4 ± 3.9		22.8 ± 4.5	27.9 ± 11.2	
Glu	80.6 ± 16.2	84.3 ± 8.8	74.5 ± 11.7		70.8 ± 5.0	79.2 ± 20.4	
Asn	62.1 ± 6.0	67.6 ± 32.2	58.8 ± 12.9		74.3 ± 13.0	92.9 ± 27.0	
Ser	214.9 ± 29.4	182.7 ± 12.3	201.5 ± 31.7		188.2 ± 16.7	207.5 ± 42.0	
Gln	909.1 ± 137.1	779.7 ± 119.0	779.0 ± 105.6		752.1 ± 82.7	834.1 ± 205.9	
Gly	157.2 ± 30.1	135.4 ± 25.2	143.2 ± 31.0		142.7 ± 23.4	159.3 ± 19.7	
Ala	514.3 ± 55.1	452.9 ± 55.4	465.3 ± 70.3		490.1 ± 38.4	578.0 ± 160.0	
Tyr	50.0 ± 8.5	42.0 ± 8.3	40.5 ± 5.6		54.0 ± 9.6	68.2 ± 15.6	
Cys	17.7 ± 9.4	14.3 ± 7.4	12.5 ± 1.7		14.8 ± 1.7	17.5 ± 5.3	
Pro	273.3 ± 50.3	244.4 ± 39.7	252.1 ± 40.9		285.7 ± 56.2	402.3 ± 160.1	
	**3 h**	**4 h**	**6 h**	**8 h**			**24 h**
His	776.7 ± 139.2	687.7 ± 109.2	809.0 ± 111.2	807.9 ± 100.9			665.3 ± 116.2
Thr	92.8 ± 36.5	73.9 ± 24.4	67.2 ± 18.8	61.8 ± 17.8			57.7 ± 20.1
Arg	122.8 ± 37.9	108.9 ± 22.4	113.3 ± 24.5	110.9 ± 9.5			112.3 ± 20.6
Val	258.3 ± 20.60 *	220.6 ± 48.1 *	191.3 ± 27.4	166.5 ± 27.7			174.3 ± 39.0
Met	40.7 ± 24.5	54.8 ± 24.5	64.9 ± 7.8	63.9 ± 12.9			51.8 ± 12.9
Trp	104.2 ± 12.94	110.8 ± 12.97	111.1 ± 27.3	98.5 ± 26.5			52.4 ± 26.6
Phe	87.5 ± 21.1 *	79.3 ± 7.2 *	74.9 ± 9.1	68.1 ± 8.4			66.2 ± 10.1
Ile	87.3 ± 43.1	77.3 ± 43.1	66.0 ± 11.0	55.7 ± 6.9			62.3 ± 6.93
Leu	167.5 ± 69.3 *	150.7 ± 69.8 *	128.7 ± 13.0	107.1 ± 16.2			101.3 ± 16.2
Lys	86.3 ± 16.1	94.4 ± 16.3	102.6 ± 68.4	93.4 ± 52.6			78.4 ± 52.6
Asp	31.3 ± 10.6	25.1 ± 7.8	29.3 ± 10.3	32.8 ± 13.6			29.0 ± 13.6
Glu	91.5 ± 40.0	70.9 ± 41.5	79.4 ± 17.5	83.1 ± 14.8			85.1 ± 14.8
Asn	91.7 ± 14.8	93.1 ± 14.8	94.7 ± 24.4	84.5 ± 14.6			58.3 ± 14.8
Ser	215.9 ± 14.66	211.7 ± 14.62	240.4 ± 45.5	238.2 ± 31.4			186.5 ± 31.4
Gln	812.7 ± 31.46	817.8 ± 31.41	831.0 ± 94.6	814.8 ± 64.2			887.3 ± 95.8
Gly	160.0 ± 95.8	171.7 ± 95.8	185.8 ± 40.3	181.7 ± 30.3			139.9 ± 30.3
Ala	538.2 ± 130.5	485.8 ± 130.4	500.1 ± 41.6	458.4 ± 38.8			489.1 ± 38.8
Tyr	71.2 ± 38.8	66.0 ± 38.8	64.9 ± 12.0	63.2 ± 11.5			56.2 ± 11.7
Cys	18.5 ± 2.3	21.0 ± 11.5	16.9 ± 2.3	16.4 ± 4.3			19.8 ± 7.4
Pro	442.9 ± 117.7	437.1 ± 117.7	442.8 ± 44.0	398.0 ± 51.3			229.9 ± 51.3

Data are shown as mean ± SD. * *p* < 0.05 *vs.* 15 and 30 min post-food intake.

Food intake is a major factor affecting PFAA levels. We previously reported that intake of 500 mg/kg glucose or D-glucosamine hydrochloride decreased canine plasma Glu, Gly, and Ala levels [19]. On the other hand, a high-protein diet increases PFAA levels [10,11,12,13,20,21]) even when measured the morning after food intake [20], indicating that plasma levels of non-essential AAs were not at all (Ser, Gln, Gly, Ala, and Cys) or only slightly (Asn, Glu, and Pro) elevated, whereas those of essential (E) AAs (The, Val, Ile, Leu, and Lys) were increased after ingestion of a high-protein meal. Val levels are also correlated with protein intake [22]. We found that plasma EAA levels were increased after food intake, with changes in Tyr, Phe, and Pro levels observed within 8 h. These results indicate that food intake must be considered for blood collection in analysis of canine PFAA levels. PFAA levels can vary according to many factors, including ingested AAs originating from dietary protein, protein digestibility, and the amount of free AAs [22,23]. However, ours is the first study demonstrating these effects in dogs. In previous human reports, it is found that PFAA levels increase after several hours of food intake [22,23]. Hutchison *et al.* reported gastrointestinal hormone, insulin, and glucagon are reacted at 40 min after whey protein intakes [24]. To our knowledge, there are few studies which investigate PFAA levels within an hour after food intake. Further studies should be conducted on the effect on homeostasis of acute load-dependent effects of food intake. Plasma Tyr and Phe levels are increased by the intake of food, especially protein. For example, plasma and brain levels of branched chain and aromatic AAs, as well as those of other AAs such as Pro, were increased by food intake in rats [25]. These changes may affect brain metabolism, including serotonin and catecholamine synthesis [11]. Our results suggest that this could occur after a single intake of food.

PFAA levels are regulated not only by food intake but also by hormones, organ metabolism, and disease [26,27]. Some reports have described alterations in PFAA levels in dogs as a result of diseases such as sepsis [14], diarrhea [28], and mammary gland tumors [6]. Further studies must be performed to investigate the relationships between PFAA levels and other factors including hormones and diseases.

More recently, more rapid and convenient methods for PFAA analysis have been developed. Joyce *et al.* also reported a method [29]. More rapid and comprehensive methods have also been recently described [30]. Their methods do not require pre-column derivatization of amino acids. The efficiency of these methods in veterinary medicine should also be investigated.

## 3. Experimental Section

### 3.1. Animals

We used five beagles (2 males and 3 females, 4–11 years old, body weight: 8–12 kg) in this study (Table 1). The animals were reared in a room with temperature and humidity maintained at 22 °C ± 2 °C and 50% ± 5%, respectively, on a 12:12-h light/dark cycle (lights on at 7:00 AM). Experimental procedures involving the dogs were approved by the Animal Research Committee of Tottori University.

### 3.2. Sample Collection and Processing

Dogs were fed commercial food (70 kcal/kg body weight; Cainz Co., Honjo, Japan) until 7:00 PM. The food consisted of 1380.2 kJ of energy (per 100 g), with 22% of the energy provided by protein, 22% as fat, and 56% as carbohydrates. Blood samples (2 mL) were initially collected at 9:00 AM (pre; day 1); thereafter, dogs were fed commercial food (50 kcal/kg body weight), and blood (2 mL) was collected 0.25, 0.5, 1, 2, 3, 4, 6, and 8 h after food intake. After all blood samples were obtained on day 1, dogs were fed commercial food (20 kcal/kg body weight). The following day (24 h after food intake) and on days 7 and 14 (hereafter referred to as days 2 and 3, respectively), blood was collected at 9:00 AM. Dogs had free access to fresh water during the experimental period.

Blood was obtained from the jugular vein and fed into tubes containing heparin and immediately separated by centrifugation at 1700 × *g* for 10 min at 4 °C. Plasma was promptly removed and frozen at –80 °C until PFAA measurements. The plasma was deproteinized in methanol (plasma:methanol (*v*/*v*) = 1:9) for 20 min; samples were then centrifuged at 15,000 × *g* for 10 min at 4 °C, and precipitated proteins were removed.

### 3.3. PFAA Measurements

All regents, including standard AA solutions (type H), were purchased from Wako Pure Chemical Industries (Osaka, Japan). We used a LC system with automated Pre-column derivatization functionality (Nexera X2; Shimadzu, Kyoto, Japan). The following basic AAs and related molecules (a total of 20 compounds) were measured and used in the analysis: Ala, arginine (Arg), Asn, cysteine-cysteine (Cys-Cys), glutamic acid (Glu), Gln, glycine (Gly), histidine (His), isoleucine (Ile), leucine (Leu), lysine (Lys), methionine (Met), phenylalanine (Phe), Pro, Ser, Thr, tryptophan (Trp), tyrosine (Tyr), and valine (Val). Standard AA solution in this study was prepared by standard AA solution (type H), Asn, Gln, Trp and Orn were diluted 5% of HCl (each concentration of AA is 250 µmo/L.

A summary of the labeling of amino acids is shown in Figure 1. At first, 0.1 mol/L boric acid solution was prepared by dissolving boric acid (0.31 g) and NaOH (0.1 g) in diluted water (50 mL). 3-Mercaptopropionic Acid (MPA) solution were prepared by dissolving 10 µL of MPA in boric acid solution (10 mL). *O*-phthalaldehyde (OPA) solution was prepared by dissolveing OPA (10 mg) in ethanol (0.3 mL), boric acid solution (0.7 mL) and diluted water (4 mL). 9-fluorenylmethyl chloroformate (FMOC) solution was prepared by dissolving FMOC (10 mg) in acetonitrile (25 mL). We used two mobile phase solution. One was 15 mmol/L potassium dihydrogenphosphate (KH_2_PO_4_) and 5 mmol/L dipotassium hydrogenphosphate (K_2_HPO_4_), which was prepared using dissolved KH_2_PO_4_ (2.04 g) and K_2_HPO_4_ (0.87 g) in diluted water (1000 mL). The other was a mixture of acetonitrile/methanol/diluted water (45/40/15). The flow rate of the mobile phase solution was 0.8 mL/min. Injected volume was 1 µL. The temperature of the column oven was 35 °C. The condition of fluorescence detection was 350 (Ex.) nm and 450 nm (Em.). LabSolutions LC/GC (Shimadzu) was used as a work station. Each PFAA level was detected according to the standard amino acid solution. Plasma levels of AAs are expressed in µmo/L.

### 3.4. Statistical Analysis

Data are expressed as mean ± SD. Differences between groups were evaluated by one-way analysis of variance followed by Tukey-Kramer’s test. A *p*-value < 0.05 was considered statistically significant.

## 4. Conclusions

Canine PFAA levels were measured by pre-column labeling LC. This method shortened the analysis time, and there were no diurnal fluctuations in most PFAA levels when measurements were made at 9:00 AM. Food intake increased the levels of a subset of AAs including some EAAs as well as Tyr, Phe, and Pro. Our results indicate that the time of day and interval after food intake must be considered when measuring canine PFAA levels.
